# About the Relevance of Triboelectric Effects and Conductive Particles in Nanogenerators Based on Cellulose Materials and Their Composites

**DOI:** 10.3390/polym18060762

**Published:** 2026-03-20

**Authors:** Ivan Muñoz, Franck Quero, Francisco Fernández-Gil, Jorge Maureira, Nicolás Rosales-Cuello, Humberto Palza

**Affiliations:** 1 Departamento de Ingeniería Química, Biotecnología y Materiales, Facultad de Ciencias Físicas y Matemáticas, Universidad de Chile, Santiago 8320000, Chile; ivan.munoz.r@ug.uchile.cl (I.M.); fquero@ing.uchile.cl (F.Q.); francisco.fernandez@ufrontera.cl (F.F.-G.); jorge.maureira.u@gmail.com (J.M.); nicolas.rosales.c@gmail.com (N.R.-C.); 2 Departamento de Ingeniería Mecánica, Facultad de Ingeniería y Ciencias, Universidad de La Frontera, Temuco 4780000, Chile; 3 Departamento de Odontología Integral Adultos, Facultad de Odontología, Universidad de La Frontera, Temuco 4780000, Chile; 4 IMPACT, Center of Intervention Medicine for Precision and Advanced Cellular Therapy, Las Condes, Santiago 7820000, Chile

**Keywords:** cellulose nanogenerator, triboelectricity, nanocomposite

## Abstract

Cellulose is a well-known biopolymer with excellent properties for a broad range of applications, including piezoelectricity for the development of nanogenerators. However, similar to other piezoelectric materials, the voltage outputs currently reported from cellulose-based piezoelectric nanogenerators (PENGs) could be overestimated due to the appearance of triboelectric processes. To understand the appearance of both phenomena, cellulose films and aerogels that had undergone several modifications to improve their piezoelectric behavior (i.e., thermal treatment and presence of piezoelectric/conductive particles) were developed and characterized. Our results show that these modifications significantly changed the dielectric properties (*ε*) and the piezoelectric coefficients (*d*_33_), with increments as high as a factor of 4, although without a clear tendency regarding the sample characteristics. Under finger-tapping mechanical stimulation, nanogenerators (NGs) using pure cellulose films generated 6 V, whereas the modified cellulose films and aerogels either increased or decreased this value, with outputs between 2 and 10 V. Notably, ternary composites, having both conductive and piezoelectric ZnO particles, increased the generation up to 24 V. There was no correlation between the voltage generated and *d*_33_ or *d*_33_/*ε* values, although some relationship with *ε* was observed, meaning that a pure piezoelectric phenomenon was not observed. This lack of correlation and the drastic decrease in the voltage generated (around 0.2 V) after changing the NG configuration show that a triboelectric phenomenon from the multilayered structure significantly contributes to the voltage generation from cellulose samples.

## 1. Introduction

Nanogenerators (NGs) emerged as a proper transducer technology able to directly convert mechanical stimuli (i.e., those coming from biomechanical movements, wind, and waves, among others) into an electrical output for energy harvesting or sensors [1]. The impact of NGs is based on considering that they can contribute to energizing sensors and wearable devices, in addition to enabling novel sensor technologies, which are required in the new era of Internet of Things, which demands clean harvesting systems [2]. For instance, novel wearable/implantable compact devices can now be designed using mechanical energy harvesting capacities from naturally occurring biomechanical phenomena through NGs [1]. As cited by Wang, “nanogenerator is a field that uses Maxwell’s displacement current as the driving force for converting mechanical energy into electric power using either piezoelectric or triboelectric effect, whether we use nanomaterials or not” [3]. While piezoelectric nanogenerators (PENGs) are based on a phenomenon associated with the intrinsic property of some materials to create polarization under mechanical strain, triboelectric nanogenerators (TENGs) use the electrostatic charges created on contacted surfaces to create electrical potential through space variation in their arrangement [3].

Despite the advantages of PENG and TENG devices to generate electrical energy, their output (either voltage, power, current, or charge) is currently assumed to be generated from some unique transducer mechanism, especially in piezoelectric-based systems, despite the clear evidence to the contrary [4]. The main issue arises from the triboelectric process, which is a universal phenomenon between any two surfaces that are in contact, as, for instance, between the different layers forming the PENG devices under pressing stimulus [5]. PENG output is driven by electrostatic induction on conductive electrodes placed above and below the piezoelectric layer, and, therefore, any effect causing a flow of charges between electrodes, such as through the triboelectric phenomenon, can contribute to the measured output [4]. In polymer-based PENGs, in addition to friction at polymer interfaces, the triboelectric phenomenon can result from shear forces in composites and even from friction or static discharge with the device generating the mechanical stimulus, for instance, via a human finger or a rubber glove [4]. Even the insulating external layers currently used in PENG devices can further contribute to charge and voltage generation through triboelectric processes, as reported recently [6]. This confusion explains that the outputs of PENG devices can be overestimated due to the contribution from the triboelectric process not being reproducible [4].

Most TENGs and PENGs are based on non-renewable synthetic polymers that, although they can meet the demand for high-performance outputs, cannot be degraded naturally and could have an adverse effect on the environment during long-term application [7]. This issue motivated the development of PENG and TENG devices based on natural polymers that, in addition to biodegradability, are one of the most promising options for biomechanical energy harvesting and sensors due to their properties, such as non-toxicity, biocompatibility, and being harmless to the human body [8]. In this context, cellulose is highlighted not only as the most abundant natural polymer in the world, having renewability, environmental friendliness, biodegradability, good mechanical strength, high crystallinity, and superior optical characteristics, but also as a good candidate for designing PENG and TENG devices [9,10]. The high crystallization through non-centrosymmetric structures and amount of polar hydroxyl groups presented in cellulose contribute to its high number of dipoles and strong electron-donating capacity, rendering piezoelectricity and triboelectric effects [10,11]. However, the piezoelectric property of pure cellulose is low, motivating its mixing with high-piezoelectric fillers such as BaTiO_3_, MnFe_2_O_4_, and single-layered MoS_2_ nanosheets, among others [9]. These particles increase the piezoelectric output of cellulose-PENG by increasing the dielectric constant and density of oriented dipoles [11]. For instance, MoS_2_ nanoparticles were added to an oxidized nanofibrillated cellulose (TOCN) obtained by modifying its primary hydroxyl group at the C6 position with 2,2,6,6-tetramethylpiperidinyl-1-oxyl (TEMPO). These nanocomposited celluloses presented longitudinal piezoelectric constant (*d*_33_) around 160% higher than pure TOCN as well as a maximum output voltage of 4.1 V and a short-circuit current of 0.21 μA [8]. Other strategies for improving the piezoelectric performance of celluloses are based on a thermal treatment and generating porous structures [9,12]. Cellulose is further an intrinsic insulating material with good dielectric properties and a slight positive electron affinity (tribo-positive effect) that can be used as a triboelectric active layer of different TENGs for improving the output performance through several modifications [13]. All these properties allow for the development of hybrid TENG/PENG cellulose devices using, for instance, nitrocellulose nanofibril paper as the triboelectric layer and BaTiO_3_/MWCNT@bacterial-cellulose paper as the piezoelectric layer with an integrated output of 18 V [14]. While nitrocellulose is tribo-negative with strong electron-withdrawing nitro groups, pure cellulose nanofibril is slightly tribo-positive due to the intrinsic abundance of oxygen atoms and the small number of carboxylic groups [14].

While the separation of triboelectric and piezoelectric signals has been discussed in the recent literature, most studies focus on mechanical or electrical decoupling methods. The specific novelty of this work lies in investigating how standard material design strategies intended to enhance piezoelectricity inadvertently modulate the parasitic triboelectric response of the device packaging. Using cellulose as a model biomaterial, we systematically evaluate the impact of common NG-enhancing modifications, namely the addition of piezoelectric nanoparticles, conductive pathways, structural porosity, and thermal treatments. By comparing devices with and without the ubiquitous PDMS insulating layer, we provide two specific new insights to the NG field. First, we demonstrate that in encapsulated devices, traditional piezoelectric predictive parameters (such as the *d*_33_ coefficient and the *d*_33_/*ε_r_* ratio) fundamentally fail to correlate with the experimental voltage output. Second, we establish that when the triboelectric contribution from the packaging layer dominates, the voltage generation is instead directly governed by the macroscopic bulk permittivity (*ε_r_*) of the engineered composites. These findings provide critical new guidelines for the accurate design and interpretation of high-performance, biopolymer-based hybrid NG.

## 2. Materials and Methods

### 2.1. Materials

The chemical oxidation of cellulose by TEMPO was performed following a method reported earlier by Saito et al. (2007) [15]. To that end, wet eucalyptus cellulose commercial pulp having a solid content value of 34.2 ± 0.5% was kindly supplied by CMPC paper company (Nacimiento, Chile) and used as raw material. All the chemicals used were purchased from Merck (Santiago, Chile), each having a purity of at least 98%. Firstly, ~11.7 g of wet pulp (equivalent to 4 g of dried cellulose) was added to 372 mL of distiller water, to which 0.4 g of NaBr was added as well as 64 mg of 2,2,6,6-tetramethylpiperidine-1-oxyl radical (TEMPO) and 30 mL of NaClO (4.00–4.99%). The reaction was performed at room temperature under magnetic stirring at 1000 rpm, maintaining pH constant (~10) by adding dropwise amount of sodium hydroxide solution (0.5 M). The reaction was stopped as soon as the pH remained constant to a value of ~10, typically after about two and a half hours. The oxidized cellulose suspension was subsequently stored at 4 °C until further use. Cellulose nanofibers were obtained following a previous report by Fukuzumi et al. (2008) [16]. The oxidized cellulose suspension was processed using a high-shear homogenizer (T-25 Digital Ultraturrax, IKA, Wilmington, NC, USA) at 15,000 rpm for 4 min. After this, the homogenized oxidized cellulose suspension was washed three times to remove most of the excess NaBr, TEMPO, and NaClO reagents. For each washing, the reaction mixture was filtered using a 9 cm internal-diameter Büchner funnel fitted with a paper filter (retention value of 12–15 µm) of the same diameter. A filter cake containing oxidized cellulose was finally obtained upon Büchner filtration and carefully removed from the surface of the filter, added to a glass beaker, and diluted using distilled water to obtain a ~400 mL suspension having a solid content of ~0.5% *w*/*v*. Finally, suspensions with volumes of ~50 mL were processed using an ultrasonicator (Branson 250 Digital (Emerson, Missouri, St. Louis, MO, USA) sonifier with 20 kHz horn frequency and 200 W maximum power) and washed three times, as mentioned earlier, to obtain suspensions of oxidized cellulose nanofibers.

ZnO micro-rods were synthesized using a modified low-hydrothermal method, as previously reported [6]. Initially, 2.9 g of hexahydrate zinc nitrate was mixed with 300 mL of deionized water and stirred magnetically for 15 min. Then, 100 mL of deionized water containing 1.4 g of hexamethylenetetramine (HMT) was added dropwise at room temperature, followed by 30 min of stirring. The mixture was then heated at 90 °C for 4.5 h. After centrifugation, the particles were dried overnight at 80 °C and calcined at 400 °C for two hours, yielding a white ZnO powder. High-purity graphite powder was used to obtain TrGO in two steps: oxidation and thermal reduction. For the oxidation of the graphite, the Hummers and Offeman methodology was used. In short, potassium permanganate and sodium nitrate were used to oxidize the graphite (GO) powder in concentrated sulfuric acid solution, as described elsewhere. The GO powder was vacuum dried at 60 °C and then milled to obtain fine GO powder. Thermally reduced GO (TrGO) was then obtained via thermal shock at 1000 °C for 20 s in a vertical quartz reactor [17]. For MoS_2_ nanosheets, bulk MoS_2_ particles were liquid-exfoliated through sonication in the presence of chitosan [18]. The chitosan powder was dissolved in 1% acetic acid aqueous solution under mechanical stirring at room temperature for 2 h, and then the MoS_2_ bulk particles were added under stirring for a further 2 h. The dispersion was then sonicated, removing the sample for stirring (2 min) every 1 h. Afterward, the dispersion was centrifuged at 2500 RPM for 15 min, and the supernatant was centrifuged at 8000 RPM for 20 min; then, the precipitate was dispersed in ethanol (300 mL) via sonication at a wave amplitude of 40% during 1 h with cycles of 5 s (5 s on/5 s off).

### 2.2. Sample Preparation and Characterization

Dense cellulose films were prepared via solvent casting as follows. For each film, 80 mL of oxidized cellulose nanofiber suspension (0.5% *w*/*v*) was magnetically stirred at 1000 rpm for 2 h. After that, the solution was sonicated using a Sonics brand Vibra Cell sonicator (Sonics & Materials, Inc., Newtown, CT, USA) at a wave amplitude of 40% over 2 h with cycles of 5 s (5 s on/5 s off). The suspension was subsequently submitted to vacuum in a desiccator at room temperature for 3 h to remove trapped air bubbles. After this, the suspension was added to a Teflon-coated petri dish and dried at 40 °C for 15 h in a laboratory oven (Memmert Type ULE 400, Memmert GmbH + Co, Schwabach, Germany). Composite films were prepared in the exact same way, except that each type of nanoparticle was added in controlled amounts, separately, by mixing with the oxidized cellulose nanofiber suspension under magnetic stirring, followed by ultrasonication for 2 h (power of 80 W using 1 s on/2 s off pulses).

The preparation of porous cellulose films was similar to that of the dense films, except that after removing the air bubbles, the suspension was frozen at −80 °C for at least 24 h, freeze-dried (Christ Alpha 1–2 LDplus, Christ/Sigma, Osterode am Harz, Germany) for 48 h, and finally pressed (Rheinstern, Riegelsberg, Germany) at room temperature. Porous composite films were prepared similarly to the porous cellulose films, except that each nanoparticle type was added separately and in controlled amounts by mixing with the oxidized cellulose nanofiber suspension under magnetic stirring, followed by ultrasonication for 2 h (power of 80 W using 1 s on/2 s off pulses).

Finally, both types of films were heat-treated. For this purpose, samples of 2 cm × 2 cm were cut and subjected to treatment at 60 °C, 100 °C, and 150 °C in a muffle furnace.

The synthesized particles and materials were characterized utilizing techniques, such as X-ray diffraction (XRD), scanning electron microscopy (SEM), Fourier-transform infrared spectroscopy (FT-IR), impedance analysis, Berlincourt Piezo meter *d*_33_, and electrical generation.

### 2.3. Nanogenerators

For the nanogeneration characterization, a standard PENG device was constructed consisting of a dielectric (cellulose-based material with or without nanoparticles) sandwiched between two copper electrodes. In the first configuration, the device was covered with an isolation layer of PDMS that kept the system out of external influences; in the second configuration, the device was not covered with PDMS to study the triboelectric effect. The PENG was coupled to a multimeter and oscilloscope for voltage measurements. For the mechanical stimulus, the devices were subjected to human finger tapping using a nitrile glove. To ensure reproducibility and validate the quantitative comparison between different samples, the excitation was carefully controlled by a single operator, maintaining a steady tapping frequency of approximately 1.3 Hz and a consistent applied force of ~1.9 N. The reported peak-to-peak voltages represent the stable average response from multiple continuous cycles. Finger tapping allows for a closer approach to the potential use of these nanogenerators for harvesting ambient mechanical vibrations and biomechanical energies and for sensors [19,20,21,22,23].

## 3. Results

### 3.1. Sample Characterization

**Figure 1:**
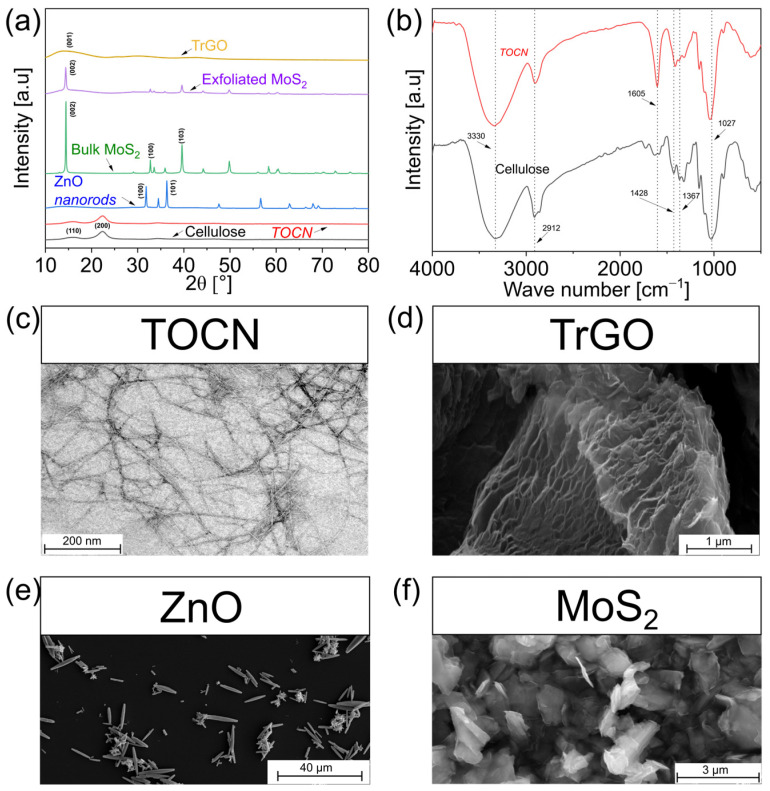
Characterization of the different particles analyzed: (**a**) X-ray diffraction pattern of pure cellulose, TOCN, ZnO nanorods, bulk MoS_2_, exfoliated MoS_2,_ and TrGO; (**b**) FTIR of pure cellulose and TOCN; (**c**) TEM image of TOCN sample; and (**d**–**f**) SEM images of TrGO, ZnO, and exfoliated MoS_2_, respectively.

The main characteristics of the different particles used (i.e., TOCN, ZnO, MoS_2,_ and TrGO) are displayed in Figure 1. The X-ray diffraction patterns of cellulose particles, displayed in Figure 1a, confirm the typical reflections of cellulose Iβ from pure cellulose and TOCN nanofibers obtained from plant cellulose [24]. Cellulose displays a reflection at 2θ~16°, corresponding to an overlapping of two peaks located at 2θ~14.9° and 2θ~16.7° from the (1–10) and (110) diffraction planes, respectively [25]. This overlapping has been reported to be related to crystallites with a diamond-shaped cross-section [26]. Another reflection was observed at 2θ~22°, corresponding to the (200) plane [25]. The TOCN sample otherwise displays a decrease in the diffraction arising from the (110) plane due to the oxidation process. Figure 1a also displays the patterns from the other particles, such as from ZnO, showing the characteristic peaks from the hexagonal piezoelectric wurtzite structure corresponding to the (100), (002), and (101) planes at 2θ = 31.7°, 34.4°, and 36.2°, respectively [6,27]. Figure 1a further confirms the exfoliation of the MoS_2_ as while bulk particles display X-ray diffractions at 14.5, 32.8, 39.8, 50.0, and 56.3°, corresponding to the (002), (100), (103), (105), and (110) planes, respectively, and exfoliated MoS_2_ nanosheets present a drastic reduction in the intensity of the (002) plane peak arising from the exfoliation process [18]. TrGO samples otherwise displayed a disordered diffraction pattern with a reduced intensity of the peak at 2θ = 26.4°, attributed to the (002) plane, confirming their thermal reduction [6]. Figure 1b displays the FTIR spectra of cellulose and TOCN nanofibers. Comparing both spectra, the most noticeable difference is the presence of an absorption peak located at ~1605 cm^−1^ for TEMPO-oxidized cellulose nanofibers. This peak corresponds to the stretching vibration of C=O belonging to aldehyde (COH) and carboxylate (COO^−^) moieties due to TEMPO oxidation [28], which is not seen in the spectra of cellulose. Regarding the morphology, Figure 1c confirms the nanosized width of the obtained TOCN nanofibers, ranging from 4 to 6 nm, as reported before by our group [29] and by others [24]. Figure 1d otherwise shows the expected porous structure of TrGO arising from the interlaminar pressure because of the liberation of gases during the thermal reduction [6,30]. ZnO nanoparticles display a hexagonal micro-rod structure, with an average diameter of 0.9 µm and an average length of 6.6 µm (Figure 1e), while exfoliated MoS_2_ particles are characterized by two-dimensional sheets with a thickness of around 100 nm and lateral sizes from several hundreds of nanometers to around 1 μm [6,31,32].

**Figure 2:**
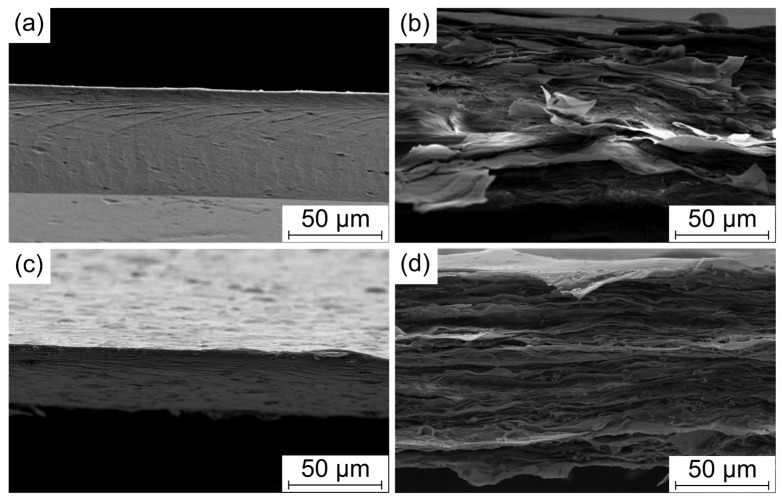
SEM images for some representative TOCN cellulose-based materials: (**a**) dense film; (**b**) aerogel film; (**c**) dense film with 2 wt% of TrGO; and (**d**) aerogel film with 40 wt% of ZnO.

Figure 2 displays SEM images of some representative films and aerogels, such as those prepared with pure TOCN cellulose nanofibers and the composites with ZnO and TrGO. The film of cellulose displayed a dense structure (Figure 2a) with a thickness of around 50 µm, while the aerogel displayed much more disordered structures (Figure 2b) and a larger thickness (around 120 µm). Rather than the standard spherical-like porous structure, the aerogel presents a layered structure arising from the collapse of the porous structure during compression after the freeze-drying process. The corresponding composites maintain the morphology of the pure samples, although the particles (or their agglomeration) can be observed at the fractured surface, as concluded from the dense film with 2 wt% of TrGO (Figure 2c) and the aerogel with 40 wt% of ZnO (Figure 2d).

### 3.2. Dielectric and Piezoelectric Behavior

**Figure 3:**
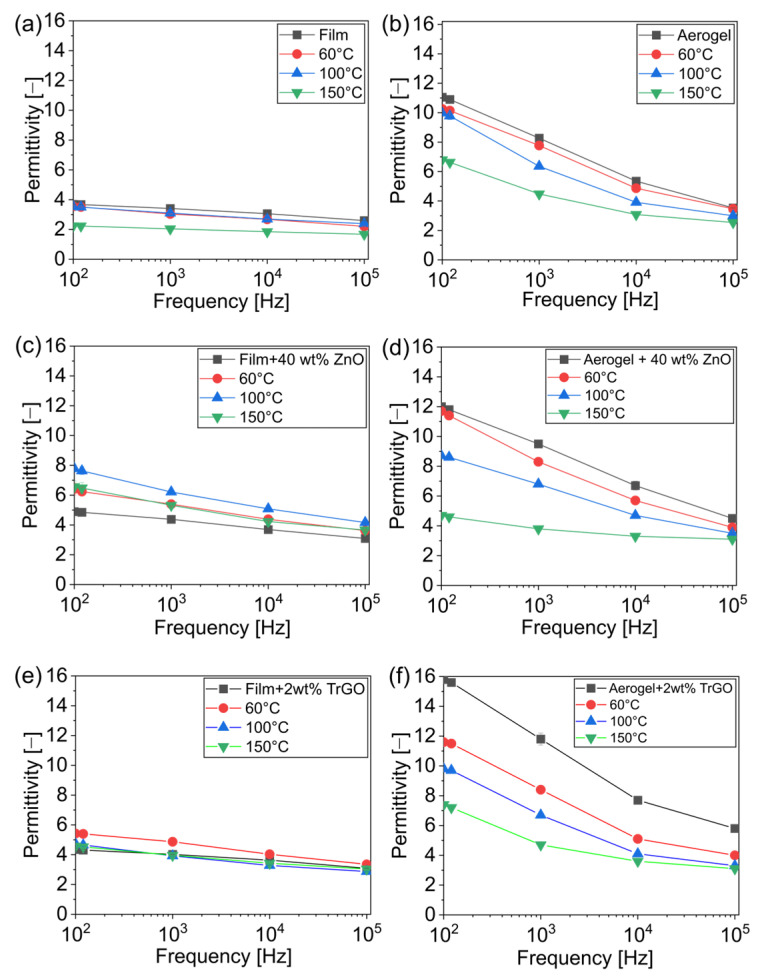
Permittivity of TOCN-based samples with different thermal treatments (60, 100 and 150 °C): (**a**) pure dense film; (**b**) pure aerogel film; (**c**) dense film composites with 40 wt% of ZnO; (**d**) aerogel film composites with 40 wt% of ZnO; (**e**) dense film composites with 2 wt% of TrGO; and (**f**) aerogel film composites with 2 wt% of TrGO.

Despite the dependence between piezoelectric nanogeneration and the dielectric properties of the piezoelectric material [6], this kind of correlation was not carried out previously in cellulose-based NG [9,33,34]. Our results confirm the large effect of pore, thermal treatment, and piezoelectric particles on the cellulose permittivity, as displayed in Figure 3 for some representative samples (see Supplementary Figure S1 for other samples). Figure 3a shows that the permittivity of pure cellulose films was barely affected by both frequency and thermal treatment, and that the sample treated at 150 °C presented lower dielectric properties, especially at low frequencies. Porous samples (Figure 3b) otherwise displayed much higher permittivity values than dense films (as high as a 250% increase) and a relevant effect of thermal treatment and frequency, decreasing their values. It is well known that the development of porous materials is a proper strategy to reduce permittivity due to the presence of air voids having lower dielectric values than the solid [35,36]. The opposite tendency found in our samples was, therefore, an unexpected result. However, it should be noted that dense films presented a residual porosity of around 16%, while porous samples presented values around 40%. The latter means a theoretical decrease in the permittivity of only 15% between dense and porous cellulose by using the Maxwell–Garnett mixing equation, assuming all the other parameters remained fixed [37]. In addition, the permittivity of a material is further related to its polarizability, dipole moment, and the number of polarized molecules per unit volume, as stated by the Debye equation applied in cellulose-based materials, and, thus, any change in the material can affect the permittivity [38]. For instance, atypically high relative permittivity in a synthesized TEMPO-oxidized cellulose paper has been found due to the directional migration of Na^+^, increased material density, and abundance of −COONa groups [24]. Indeed, the collapsed structure observed in our porous cellulose (see Figure 2b) and the same freeze-drying process involved can increase the permittivity [34]. The polymer alignment during the freeze-drying process and the pseudo-multilayer structure with different dipole orientations explain the higher permittivity, as reported for PVDF samples [39]. The high surface area in porous samples can further trigger other mechanisms explaining higher dielectric behaviors, such as an increase in the surface concentration of polar hydroxyl groups, breaking the hydrogen bond network, and the absorption of water molecules [40]. Aerogels further presented a much higher frequency sensitivity than dense samples due to the presence of more flexible surface functional groups having a relevant downward trend with the frequency from relaxation effects [41]. At low frequencies, the flexible dipole has enough time to arrange, improving the dielectric constant of the polymer, while at high frequencies, the dipoles do not have enough time to be polarized, and the dielectric constant decreases [42]. On the other hand, thermal treatment on the aerogel decreased the permittivity, likely due to a change in the chemical structure, as reported previously, in addition to chemical loss of water, both processes facilitated by the porous structure [12]. By comparing dense and porous samples, the relevance of high surface area on the effectiveness of the thermal treatment as a method to change the dielectric properties is clear.

Figure 3c,d show the effect of adding 40 wt% of ZnO on the permittivity of dense and porous films. For dense films, the piezoelectric particles increase the permittivity, although with a more relevant effect after thermal treatment, increasing their values by even 100%. In composites prepared through physical blending, the dielectric properties are mainly influenced by the charge at the interface of each phase, especially when inorganic nanofillers with high dielectric constants are used [6,40,43]. These tendencies were confirmed for composites having 2, 10, and 30 wt% of ZnO, meaning that their permittivity presented higher values than pure cellulose and that these changes were highly dependent on the filler concentration and thermal treatment (see Supplementary Figure S1). For instance, the highest values of permittivity were 14 and 12 at 10^2^ Hz for composites having 10 and 30 wt% of filler after a thermal treatment at 150 and 60 °C, respectively. It is likely that the better dispersion of nanoparticles at low concentrations can explain this tendency. Notably, this tendency is to the contrary when the particles are incorporated into the porous samples, as the permittivity is barely affected or even decreased (Figure 3d). More likely, the dielectric particles could disrupt the phenomena, explaining the higher permittivity of porous TOCN, such as the high amount of functional surface groups, polymer alignment, and even changes in the porosity. Composites having 2 wt% of MoS_2_ presented behavior similar to that of ZnO at 40 wt%, meaning a positive effect on dense films and barely any effect on porous films (see Supplementary Figure S1). Regarding the effect of conductive particles (TrGO), a relevant effect was not observed in dense samples as all composites present similar values independent of the thermal treatment (see Figure 3e for composites with 2 wt% of TrGO). In porous samples otherwise (Figure 3f), a drastic increase is observed with a large effect on the thermal treatment, with the highest values (around 16) obtained for the composited aerogels without thermal treatment. By comparing this composited aerogel with pure dense cellulose, an increase by a factor of 4 in permittivity is established. The high effect of conductive particles is currently explained by the enhancement in the interfacial polarization, for instance, through the Maxwell–Wagner–Sillar (MWS) effect, arising from charges accumulated in the polymer/particle interfaces, forming local microcapacitors [6,41,44]. As the frequency increases, the dipoles have less time to reorient themselves in response to the electric field, which results in a lower capacity to polarize and a decrease in relative permittivity [45].

**Figure 4:**
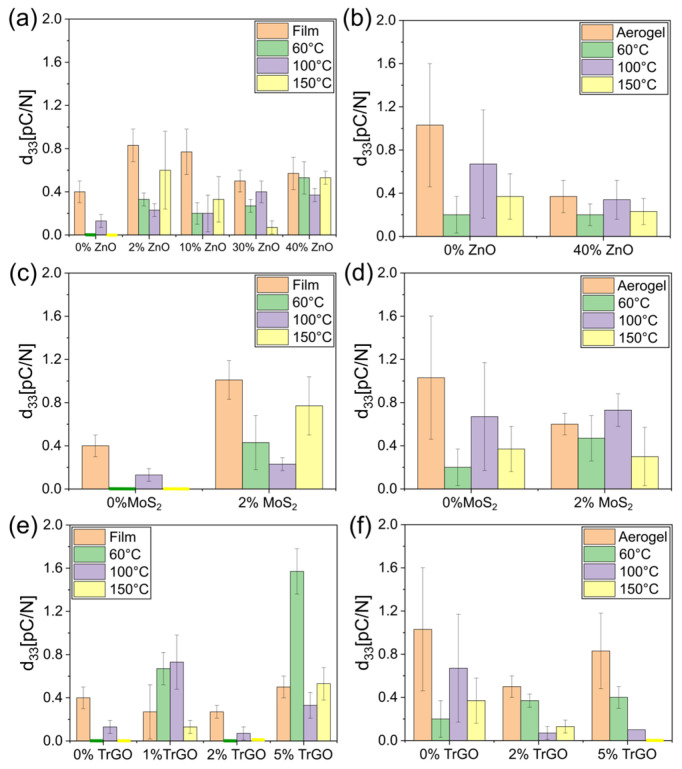
Experimental values of piezoelectric constant *d*_33_ for some representative cellulose-based samples thermally treated at 60, 100, and 150 °C: (**a**) dense films with ZnO; (**b**) aerogel films with ZnO; (**c**) dense films with 2 wt% of MoS_2_; (**d**) aerogel films with 2 wt% of MoS_2_; (**e**) dense films with TrGO; and (**f**) aerogel films with TrGO.

Figure 4 displays the piezoelectric coefficient (*d*_33_) of the most relevant samples tested. Although cellulose is a well-known piezoelectric material, its performance is highly dependent on processing and the presence of piezoelectric fillers [46]. Cellulose films presented *d*_33_ = 0.4 pC/N (Figure 4a), in agreement with previously reported values [46,47]. However, the thermal treatment reduced the piezoelectric behavior (Figure 4a), likely associated with changes in the material structure due to the temperatures used for the modifications. For instance, thermal treatment increases the crystallinity of cellulose [12,48] in addition to reducing the water content and triggering thermal degradation, lowering the number of functional groups [12]. All these changes can decrease *d*_33_, as reported previously [12,49]. Similar to other composited cellulose piezoelectric materials, the presence of ZnO drastically increased *d*_33_ (for instance, at 100% for 2 and 10 wt% of filler), depending on the thermal treatment of the cellulose (Figure 4a). However, the improvement is low compared with other reported composites, such as those based on BaTiO_3_ reaching values as high as 5 pC/N [47]. Porous cellulose presented higher *d*_33_ values than dense films (Figure 4b) due to its low density and modulus, leading to greater deformation and more uniform stress distribution [34,41]. Although thermal treatment decreases the piezoelectric coefficient in porous samples as compared with non-treated cellulose, the values obtained are higher than those of dense films for all the temperatures tested (Figure 4a). However, the composited aerogel with 40 wt% of ZnO presented a contrary tendency, decreasing *d*_33_ independent of the thermal treatment (Figure 4b). This non-expected result may be associated with the reinforcement effect of these micro-particles, increasing the stiffness of the pores and limiting the deformation. The same tendency was found for composites having 2 wt% of MoS_2_ and 1, 2 and 5 wt% of TrGO particles (Figure 4c,d and Figure 4d,e, respectively) as, in dense films, they increase *d*_33_ and, in aerogels, the effect is to decrease this property [9]. In porous piezoelectric materials, the relevance of the structure and elastic modulus of the walls on *d*_33_ has been reported [50,51]. In addition, in composited piezoelectrics, the elastic modulus of the piezoelectric filler can also reduce the *d*_33_ [52].

**Figure 5:**
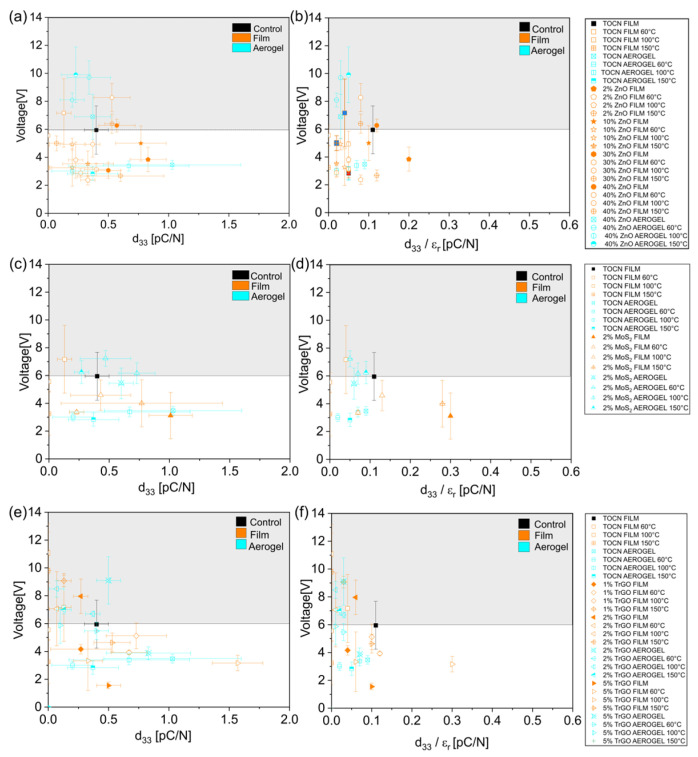
Effect of *d*_33_ and the *d*_33_/*ε_r_* ratio on the voltage generated under finger-tapping stimulus at 1.3 Hz. Grey zone represents voltages above 6 V. (**a**,**b**) for samples having ZnO particles; (**c**,**d**) for samples having MoS_2_ particles; and (**e**,**f**) for samples having TrGO. In all the figures, values for pure dense and aerogel samples treated at different temperatures are displayed for comparison. The grey- and white-colored areas represent values higher and lower than control (pure dense cellulose), respectively.

For the analysis of NG behavior using different samples, a device consisting of a piezoelectric film sandwiched between two copper electrodes and covered with two PDMS insulation layers was used. The thickness of each sample is shown in Table S1. The mechanical stimulus was carried out by pushing the sample with a human finger covered with a glove. Human finger tapping was deliberately chosen as the stimulus to accurately emulate the real-world operational environment of these flexible composites for biomechanical energy harvesting applications. Under these conditions, the pure cellulose dense film generated a peak-to-peak voltage of around 6 V, which agrees with the order of magnitude of previous results from cellulose films [46]. A direct comparison with previous PENG reports is not possible due to the different geometries of the devices, in addition to the variation in the force and frequency of the stimulus. Pure cellulose aerogel, despite its higher *d*_33_, produced a voltage of 3.5 V under the same conditions, contrary to previous reports stating that the presence of porosity could increase the generation. Therefore, in our case, the increasing material deformation under stress, which increases strain and, thus, the piezoelectric output in porous materials, can be ruled out, likely due to the collapsed structure in our sample [53]. This poor electromechanical conversion is further confirmed when normalizing the output voltage by the sample thickness (Table S1). The generated electric field of the pure dense film (~0.094 V/µm) is significantly higher than that of the pure aerogel (~0.021 V/µm), validating that the intrinsic piezoelectric response is lower in the porous structure. Regarding thermal treatment of dense films, there is an effect, although without a clear tendency, as the sample treated at 100 °C showed 7.0 V of generation, while the samples treated at 60 and 150 °C presented values of 5.8 and 3.2 V, respectively. No significant effect of the thermal treatment was established for aerogels. A similar lack of tendency is displayed from the composites with ZnO, although there is a clear improvement in samples having 40 wt% of filler with generations of around 10 V for aerogels treated at 100 and 150 °C (nearly three-times higher than pure aerogel) and around 8 V for aerogels treated at 60 °C and dense films treated at 60 °C. However, some composites presented lower values, for instance, 2.0 V for dense films having 2 wt% of ZnO treated at 60 and 150 °C. A summary of the voltage generated and the associated *d*_33_ values for all the samples is displayed in Figure 5a.

Despite the direct relationship between the voltage generated in PENG and the *d*_33_ of the piezoelectric material, as stated by the direct piezoelectric phenomenon, Figure 5a does not display any tendency between these two variables. However, the standard equation for PENG from an equivalent capacitive circuit states the following [54]:

$$V=\frac{d_{33}\cdot F}{C_{PENG}}\;\propto \frac{d_{33}}{\varepsilon_{r}}$$

where *F* is the applied force, and *C_PENG_* is the capacitance of the PENG, which is linearly related to the permittivity of the system (*ε_r_*). This expression stresses that generation is not only related to the piezoelectric coefficient *d*_33_ but rather to the *d*_33_*/ε_r_* ratio [54]. Figure 5b displays the effect of this ratio on the voltage generation, where a clear tendency was again not observed, confirming that our results did not fit PENG theories. Notably, the same lack of tendency was further observed in samples with MoS_2_ (Figure 5c,d) and TrGO (Figure 5e,f). In Supplementary Figure S2, the values of peak-to-peak voltages of representative samples are displayed together with the electrical current obtained simultaneously (around 0.2 µA), in addition to the resulting power generation (around 1.0 µW). From this figure, it is concluded that changes in the current obtained from the different samples exhibit the same trend as the generated voltages.

To complement the results from the NG under finger-tapping stimulus at 1.3 Hz, an external force testing device operated at 35 Hz by applying a similar force was further used for the mechanical stimulus, as displayed in Supplementary Figure S3. These results show that by changing the mechanical stimulus (frequency and contact area), the nanogenerator output is largely affected. In particular, while, under these conditions, the voltage output decreased to values lower than those obtained via finger-tapping stimulus (below 2 V for all the samples) due to an increase in the stimulated area, the current increased due to the higher frequency used. The increase in the electrical current with the frequency is a well-known correlation displayed by both piezoelectric and triboelectric NG, explained by changes in the impedance, resistance, or capacitance of the system and the increase in charges generated per unit of time [55,56]. Therefore, these results do not permit a definitive conclusion about the mechanism behind the generation observed in our cellulose-based samples. Indeed, Figure S4 shows a summary of the voltage generated and the associated *d*_33_ values for the samples, confirming that a tendency between these two variables was not found at higher frequency.

**Figure 6:**
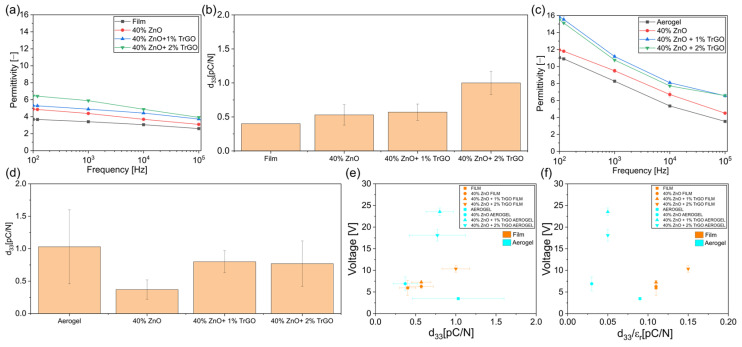
Effect of a conductive particle (TrGO) on the dielectric and piezoelectric properties of cellulose composites with 40 wt% of ZnO: (**a**) permittivity and (**b**) piezoelectric constant for dense films; and (**c**) permittivity and (**d**) piezoelectric constant for aerogel films. Voltage generation under finger-tapping stimulus at 1.3 Hz from the hybrid samples against (**e**) the *d*_33_ and (**f**) the *d*_33_/*ε_r_* ratio.

To further advance our analysis, the effect of conductive nanoparticles on the behavior of piezoelectric cellulose was analyzed. The addition of electrically conductive particles to piezoelectric composites is a proper strategy to enhance charge distribution as they can collect charges from the piezoelectric particles during deformation, thereby improving the overall piezoelectric response, as shown in piezoelectric polymer/ZnO composites with TrGO [6]. To study this effect, composites based on cellulose with 40 wt% of ZnO were prepared with 1 and 2 wt% of conductive TrGO. Figure 6a displays the permittivity of the ternary dense composites, showing that conductive particles enhanced the dielectric properties mainly due to the interfacial polarization (Maxwell–Wagner–Sillars effect) and the electron migration near conductive particles forming dipoles [6]. The presence of conductive particles further increased the piezoelectric constant of dense cellulose (Figure 6b) as they can generate electrically conductive pathways that enhance electron flow and induce additional polarization [6]. In aerogels, conductive particles not only increase the permittivity (Figure 6c) but also compensate for the drop in the *d*_33_ from the composite, increasing its value (Figure 6d). Notably, in dense ternary composites, the voltage generation is barely affected, while in aerogels, the voltage increases to values as high as 24 V for a sample with 1 wt% of TrGO. Figure 6e,f confirm the lack of a proper tendency regarding the dependence between the voltage generation and *d*_33_ and *d*_33_/*ε* in all ternary composites.

### 3.3. Triboelectricity

**Figure 7:**
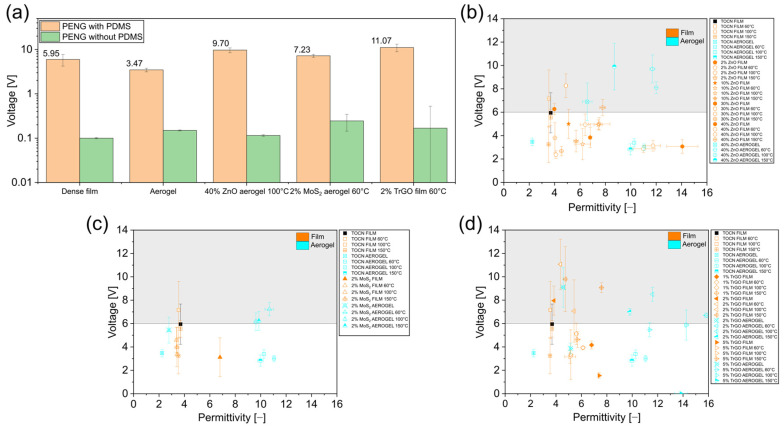
(**a**) Effect of the presence of a PDMS insulating layer on PENG generation for some representative samples. Effect of the permittivity on the voltage generated for cellulose samples: (**b**) ZnO particles; (**c**) MoS_2_ particles; and (**d**) TrGO particles. In (**b**–**d**), the figures’ values for pure dense and aerogel samples treated at different temperatures are displayed for comparison. The grey- and white-colored area represents values higher and lower than the control (pure dense cellulose).

Our results show that, although cellulose can increase the voltage generation by adding piezoelectric particles (such as ZnO) and through the presence of conductive TrGO particles in ternary composites, the output is not related to the two main parameters for piezoelectric generation: *d*_33_ and *d*_33_/*ε_r_*. Based on previous antecedents showing that triboelectricity is a universal phenomenon, whenever two surfaces are in contact, the effect of the isolated PDMS layer on our NG behavior was analyzed, as it can generate triboelectric signals in multilayered devices [5,57]. By knowing that friction at polymer interfaces in NG devices can generate triboelectricity, we tested some representative samples (pure cellulose film, pure cellulose aerogel, composited aerogel with 40 wt% of ZnO thermally treated at 100 °C, composited aerogel with 2 wt% of MoS_2_ thermally treated at 60 °C, and a dense film composite with 2 wt% of TrGO thermally treated at 60 °C) using devices without the isolated PDMS layer (see Figure 7a) [4]. The voltages generated under these conditions were at least one order of magnitude lower than those obtained from a PENG constructed with a PDMS isolation layer, showing the relevance of triboelectric phenomena from the interlayer contact between PDMS and the electrode. It is relevant to note that PDMS is a well-known negative triboelectric layer, so its contact with the electrode in our NG configuration can in situ generate a single-electrode TENG [57,58]. Crucially, because the triboelectric generation is primarily governed by the contact between the uniform PDMS layer and the electrode, the effective contact area remains constant across all measurements. Consequently, the variations in output voltage between dense films and highly porous aerogels cannot be attributed to differences in their internal surface area. Instead, as discussed below, these variations are driven by how the different morphologies and fillers modify the bulk dielectric properties of the device. Based on these findings, we further extend our analysis regarding the dependence of the voltage generation on some sample characteristics, in addition to *d*_33_ and *d*_33_/*ε*. In particular, there is open discussion about the effect of the dielectric properties on the output of TENG [59]. Despite some theories supporting the idea that lower-dielectric-constant media result in higher TENG output, several experimental studies showed that by increasing the dielectric constant of triboelectric surfaces, an increase in the TENG output power is produced [59]. Motivated by this discussion, we plotted the output voltage from our generators against the permittivity, as displayed in Figure 7b–d for samples with ZnO, MoS_2,_ and TrGO, respectively. Obviously, the trend observed from these figures did not allow for any conclusion regarding a direct relationship between the permittivity and the generation, but at least there is an improvement in the dependence as compared with the figures plotting the generation against *d*_33_ and *d*_33_/*ε* (see Figure 5). For instance, in Figure 5, most samples with higher generation than control (pure dense cellulose film) presented equal or lower *d*_33_ and *d*_33_/*ε*, while in Figure 7, the opposite was observed. To further validate the relevance of the layers presented in NG on the generation, we tested an NG of pure cellulose film as dielectric with an extra layer of carbon film. The voltage generated with this carbon/PDMS/electrode/cellulose/electrode/PDMS/carbon structure was 1.6 V, confirming that the generation was not fully associated with the piezoelectricity of the cellulose but rather with the presence of the external film in contact with the finger actuator. In this case, the conductive external layer avoids all the triboelectric charges generated from PDMS moving to the copper electrode, reducing the voltage measured.

**Figure 8:**
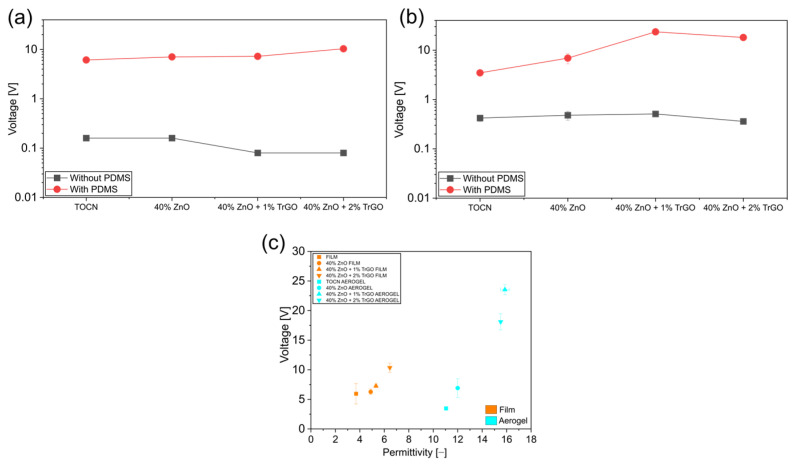
(**a**,**b**) Effect of the presence of a PDMS insulating layer on PENG generation for ternary composites based on cellulose with 40 wt% of ZnO and TrGO, for dense and aerogel samples, respectively; (**c**) effect of the permittivity on the voltage generated for cellulose ternary samples.

The same tendency regarding the large triboelectrical effect of the PDMS insulating layer was established for ternary composites (Figure 8a,b), with both dense films and aerogels showing one order of magnitude lower generations after removing this layer than NG with PDMS. Notably, in this case, a clear direct relationship was established between the voltage generation and the permittivity of the samples (Figure 8c). Therefore, a clear triboelectric contribution to the generation of our NG can be concluded. A similar tendency regarding the relevance of triboelectricity in PENG was recently reported in polycaprolactone composites with ZnO piezoelectric particles [6].

## 4. Conclusions

By testing cellulose materials with several modifications to improve their piezoelectric behavior, such as thermal treatment, porous structure, piezoelectric nanoparticles, and conductive particles, relevant conclusions were obtained regarding nanogeneration. Under controlled finger-tapping mechanical stimulation, pure cellulose films generated ~6 V, while modified films and aerogels exhibited varying outputs between 2 and 10 V. Noteworthily, ternary composites incorporating both piezoelectric (ZnO) and conductive (TrGO) particles significantly increased the generation up to 24 V. Crucially, the voltage generated across this wide array of samples did not follow any correlation with standard piezoelectric predictive parameters, such as the *d*_33_ coefficient or *d*_33_/*ε* ratio, ruling out a pure piezoelectric contribution to the generation. Instead, our findings experimentally demonstrate that the ubiquitous PDMS isolation layer used in standard NG devices acts as a primary active surface for triboelectric generation. By removing this packaging layer, the voltage output dropped by an entire order of magnitude. Most importantly, we establish that in these triboelectrically dominated hybrid devices, the output performance is directly governed by the macroscopic bulk permittivity of the engineered composites, rather than their piezoelectric constants. Therefore, structural and compositional modifications enhance the output not by boosting the intrinsic piezoelectricity but by increasing the dielectric permittivity of the bulk material, which, in turn, maximizes the capacitive TENG effect. Based on these findings, we conclude that unrecognized triboelectric phenomena likely account for many of the exceptionally high voltage generations previously reported in cellulose-based PENGs. This insight provides a vital new perspective for the accurate design, evaluation, and signal separation of flexible nanogenerators across a wide range of active materials.

## Data Availability

The original contributions presented in this study are included in the article and Supplementary Material. Further inquiries can be directed to the corresponding author.

## Supplementary Materials

The following supporting information can be downloaded at: https://www.mdpi.com/article/10.3390/polym18060762/s1, Figure S1. Permittivity of TOCN-based samples with different thermal treatments (60, 100 and 150 °C): (a) dense film composites with 2.0 wt% of MoS2; (b) composite aerogel with 2 wt% of MoS_2_; (c) dense film composites with 2 wt% of ZnO; (d) dense film composites with 10 wt% of ZnO; (e) dense film composites with 30 wt% of ZnO; (f) dense film composites with 40 wt% of ZnO; and (g) dense film of pure TOCN. Figure S2. Voltage, current and power generation from representative samples under finger-tapping stimulus: (a) films treated at 100 °C with ZnO filler; (b) aerogels treated at 100 °C with ZnO filler; (c) films treated at 60 °C with 2 wt% of MoS_2_; (d) aerogels treated at 60 °C with 2 wt% of MoS_2_; (e) films treated at 60 °C with TrGO filler; and (f) aerogels treated at 60 °C with TrGO filler. Figure S3. Voltage, current and power generation from representative samples under an external force testing device for the mechanical stimulus at 35 Hz: (a) films treated at 100 °C with ZnO filler; (b) aerogels treated at 100 °C with ZnO filler; (c) films treated at 60 °C with 2 wt% of MoS_2_; (d) aerogels treated at 60 °C with 2 wt% of MoS_2_; (e) films treated at 60 °C with TrGO filler; and (f) aerogels treated at 60 °C with TrGO filler. Figure S4. Effect of *d*_33_ on the voltage generated under an external force testing device for the mechanical stimulus at 35 Hz. Table S1. Average thickness of the different samples used for the fabrication of nanogenerators.
