# Supplementary material for: About the Relevance of Triboelectric Effects and Conductive Particles in Nanogenerators Based on Cellulose Materials and Their Composites
Source: Polymers (Basel). 2026 Mar 20;18(6):762. doi: 10.3390/polym18060762 (PMC13030570; doi:10.3390/polym18060762)
Supplement: Supplementary file 1 [file polymers-18-00762-s001.zip › polymers-4161227-supplementary.pdf]

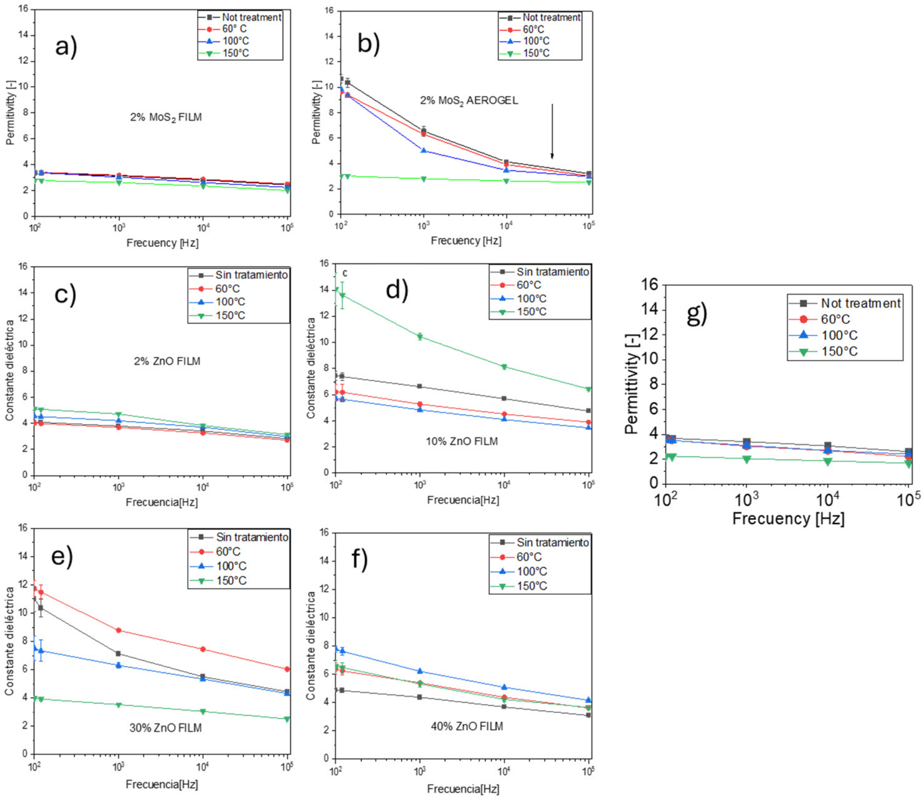

Figure S1. Permittivity of TOCN based samples with different thermal treatments (60, 100 and 150 °C): a) dense film composites with 2.0 wt% of MoS_2 ; b) composite aerogel with 2 wt% of MoS_2; c) dense film composites with 2 wt% of ZnO; d) dense film composites with 10 wt% of ZnO; e) dense film composites with 30 wt% of ZnO; and f) dense film composites with 40 wt% of ZnO; and g) dense film of pure TOCN.

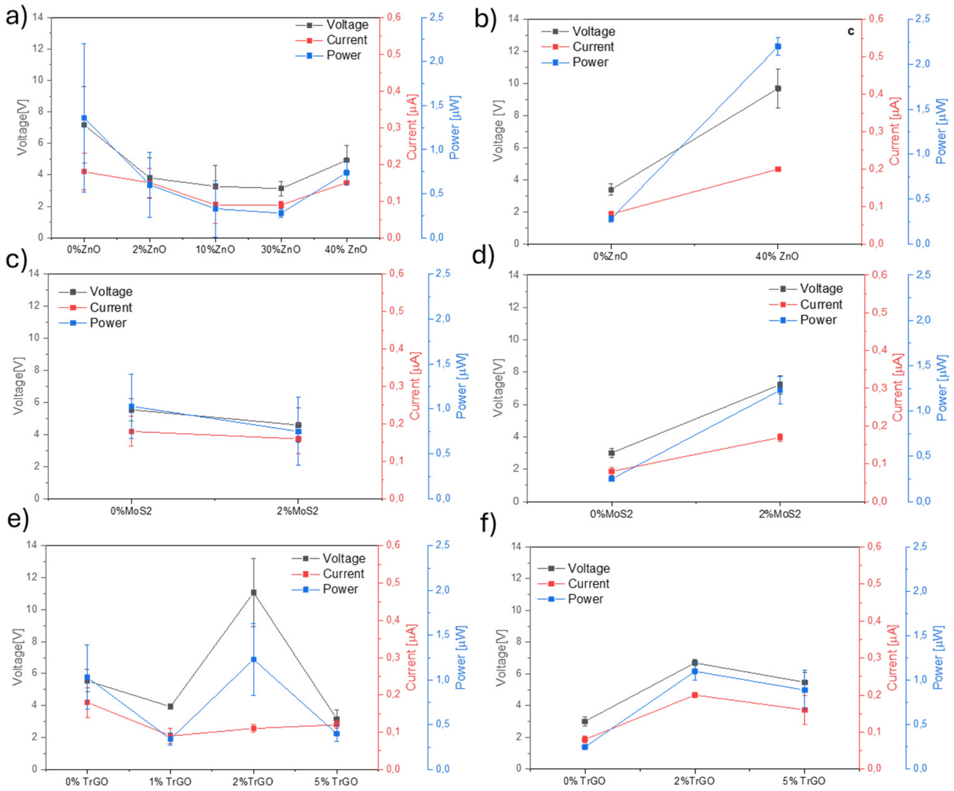

Figure S2. Voltage, current and power generation from representative samples under finger-tapping stimulus: a) films treated at 100 °C with ZnO filler; b) aerogels treated at 100 °C with ZnO filler; c) films treated at 60 °C with 2 wt% of MoS_2; d) aerogels treated at 60 °C with 2 wt% of MoS_2; e) films treated at 60 °C with TrGO filler; and f) aerogels treated at 60 °C with TrGO filler.

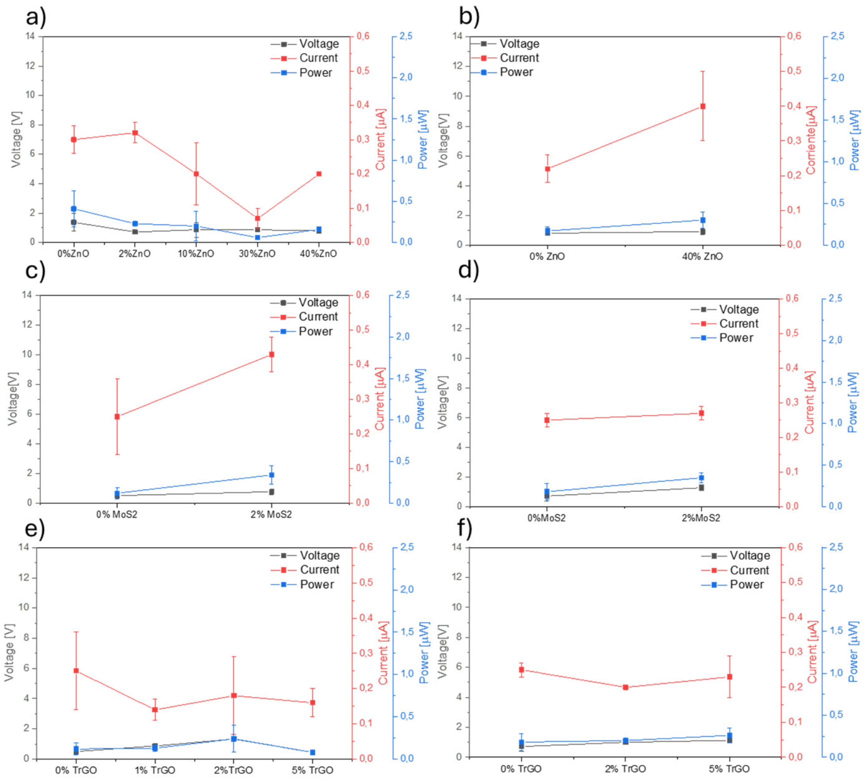

Figure S3. Voltage, current and power generation from representative samples under an external force testing device for the mechanical stimulus at 35 Hz: a) films treated at 100 °C with ZnO filler; b) aerogels treated at 100 °C with ZnO filler; c) films treated at 60 °C with 2 wt% of MoS_2; d) aerogels treated at 60 °C with 2 wt% of MoS_2; e) films treated at 60 °C with TrGO filler; and f) aerogels treated at 60 °C with TrGO filler.

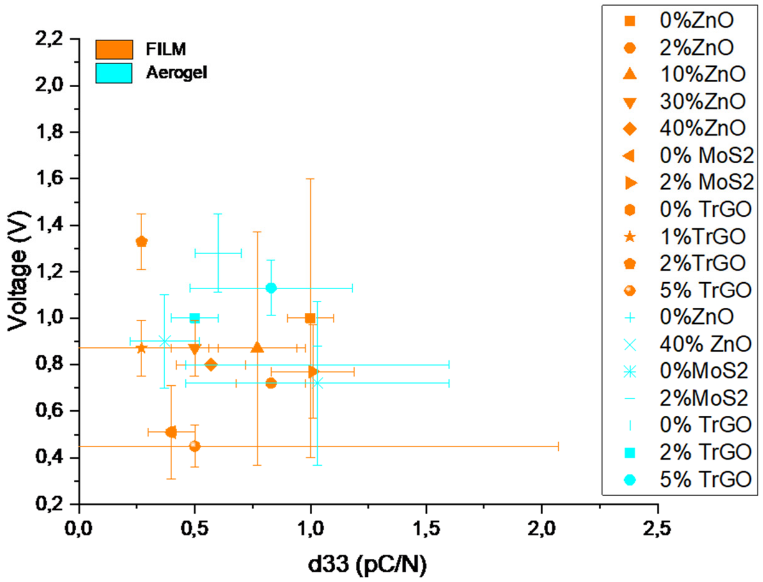

Figure S4. Effect of d33 on the voltage generated under an external force testing device for the mechanical stimulus at 35 Hz.

Morphology	Specimen	Average Thickness (μm)
Film	TOCN	63.8
Film	ZnO Composite	82.1
Film	MoS2 Composite	59.5
Film	TrGO Composite	89.1
Aerogel	TOCN	165.3
Aerogel	ZnO Composite	159.4
Aerogel	MoS2 Composite	180.4
Aerogel	TrGO Composite	187.7

Table S1. Average thickness of the different samples used for the fabrication of nanogenerators.
